# Architecture that Might Have Contributed to Disease Prevention

**DOI:** 10.3201/eid3006.AC3006

**Published:** 2024-06

**Authors:** Terence Chorba

**Keywords:** Tuberculosis, tuberculosis and other mycobacteria, diabetes mellitus, Samoa, American Samoa, architecture, fale, art and science, About the Cover

**Figure:**
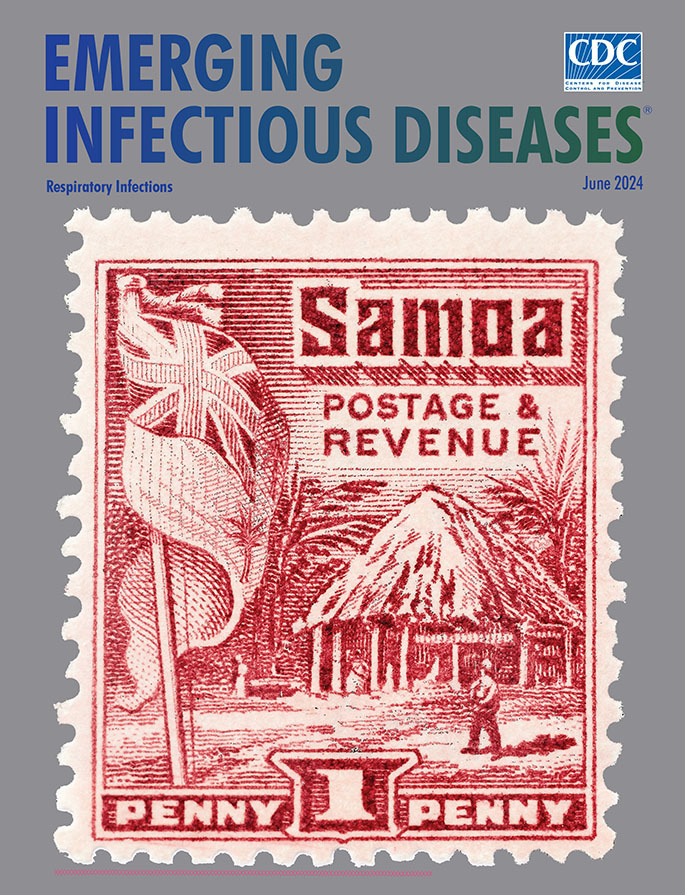
**J. Potts (details unknown), engraver for Bradbury, Wilkinson & Co. Printer. Fluttering New Zealand flag and Samoan house on stamp with designation “Samoa,” by civil administration of the mandated territory of Western Samoa, 1921.** Printed by Government Printing Office, Wellington, New Zealand. Private collection, Atlanta, Georgia. Photography by Will Breedlove.

The Samoan Islands constitute a volcanic archipelago covering 1,170 square miles of the central South Pacific Ocean in the mid-Western part of the Polynesian triangle. The Islands are divided into 2 administrative jurisdictions—the Independent State of Samoa in the west and American Samoa in the east—separated by approximately 40 miles of water. Evidence of human occupation dates back 3 millennia, but European exploration to the islands was first documented in the mid-18th century. In December 1899, the Samoan archipelago was formally partitioned into a German colony (German Samoa) in the west and a US territory (American Samoa) in the east. During World War I, New Zealand forces overtook the western islands, and in 1920, German interests were formally surrendered to New Zealand, which granted political independence to the western jurisdiction in 1962. In addition to English, the indigenous Polynesian people of 2 jurisdictions share a common Polynesian language, Samoan; in both jurisdictions, most Samoans are exclusively of Samoan ancestry.

As for many other Pacific Island jurisdictions, prevalence rates for adult-onset diabetes among the inhabitants of the Samoan Islands are among the highest in the world. In 2013, population-based surveys in the Independent State of Samoa found that, among adults (25–64 years of age), burdens of type 2 diabetes (commonly assessed in untreated persons as hemoglobin A1C >6.5% or fasting blood sugar >126 mg/dL) were 19.6% among men and 19.5% among women. Many observational cohort studies of persons with latent tuberculosis infection (LTBI) have found a higher risk for tuberculosis (TB) disease—generally 3-fold— among those with diabetes than among those with normal blood sugar levels. In most other Pacific Island jurisdictions where rates of type 2 diabetes are high, rates of TB are also high. For example, in the Federated States of Micronesia, TB incidence rates (cases/100,000 population) of 73.7 were reported in 2020 and 62.8 in 2021. By contrast, the World Bank lists the Independent State of Samoa as a low TB incidence jurisdiction with a reported TB incidence rate of 6.8 in 2021, comparable to that of New Zealand. In American Samoa, similarly low TB incidence rates of 6.8 were reported in 2020 and 8.6 in 2021. Although reasons to account for the disparity in TB incidence between the Samoan and the other Pacific Island populations may be matters of speculation, the architectural structure on the Samoan stamp on the cover of this month’s journal may be in part responsible.

**Figure 2:**
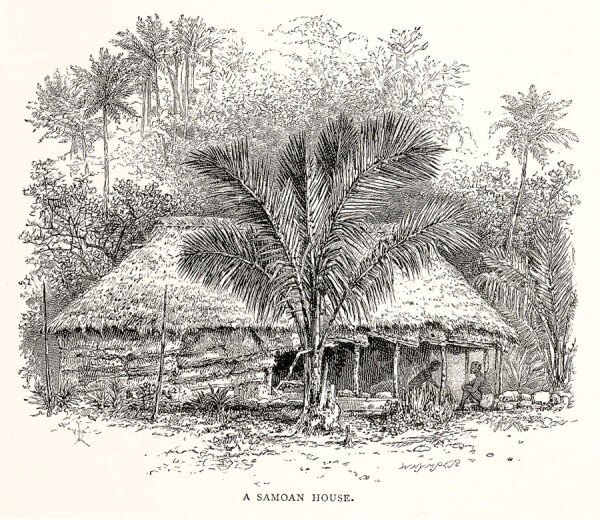
A late 19th-century artist’s depiction of a standard fale, showing roll-down screens or weather blinds, called pola.

Among the shared cultural features of the Samoan peoples is the basic design of a house, or fale (pronounced fah-leh), the Samoan word referring to houses of any size, including traditional housing structures, workplaces, and meetinghouses. Although found elsewhere in Polynesia as well, the architecture of the fale is a source of great pride to Samoans and is a characteristic art form often featured in representations of Samoan life and culture. A traditional fale has an oval shape; a domed roof supported by breadfruit, coconut, or poumuli wood posts; and no permanent or structural walls (Figures 1, 2). Roll-down screens or weather blinds, called pola, are often fashioned and hung between the external wooden posts to afford some shelter and privacy, over a raised stone floor. Before the arrival of the European powers and availability of imported materials, metal and structural walls were not used in fale construction, in which beams and posts would be fastened together and plaited coconut fiber rope would be used for lashing. Roofing on top of a domed framework was thatch made of leaves of lau (taro), coconut, or sugar cane. This author would like to advance the theory that, although all modern-day houses in the 2 Samoas are constructed with walls, the fale has, in part, played a positive role in reducing the spread of TB infection in the Samoan population.

**Figure 3:**
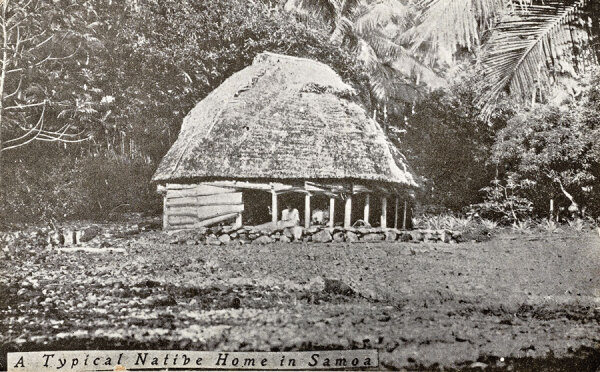
An early 20th-century photograph of a fale, a thatched pavilion without walls.

Throughout the world, almost all TB transmission results from a person inhaling droplet nuclei containing *Mycobacterium tuberculosis*, the dehydrated residua of larger respiratory droplets generated by persons with pulmonary or laryngeal TB infection who are coughing, sneezing, or otherwise aerosolizing the infecting bacterium. The odds of inhaling such particles are a function of how long the particles can remain suspended in the ambient air and follow room currents. After *M. tuberculosis* organisms strike surfaces, it is very difficult for them to re-aerosolize from droplet nuclei as respirable particles. A leading contributor to increased pulmonary infections in temperate climates during winter and in tropical climates during rainy seasons is the increased time that most people spend indoors, putatively sharing airspace with others who already are infected and infectious.

One outstanding meteorologic feature of the Samoan Islands is the often-present wind; Southeast trade winds bring clouds and rain throughout the year. In Western Samoa, the highest average wind speeds are 11.9 miles/hour from June through October, and the lowest average wind speeds are 9.7 miles/hour in March. Unlike the common rectangular domiciliary and institutional structures found in other Pacific Island groups and introduced to the Samoan Islands by the Europeans in the 19th century, often with airtight or insulated building shells, the traditional fale both lacking walls and being buffeted consistently by wind would provide housing space that was naturally ventilated and cooled. Such structures would also not provide an environment conducive to person-to-person spread of respiratory particles. If this has been the case among Samoan populations for centuries, one would expect modern-day LTBI prevalence and TB disease incidence to be low and TB transmission to be rare among Samoans relative to other Pacific Island peoples. Unfortunately, due to lack of medical care access and lack of diagnostics, no systematically gathered rates of TB infection or disease are available for the Pacific Island populations from the first half of the 20th century. Recent World Health Organization estimates for the many Pacific Island jurisdictions show a wide range of TB incidence, no doubt the results of a multiplicity of factors; however, few Pacific Island jurisdictions report low TB incidence rates comparable to those of the Samoas (e.g., Tonga and the Cook Islands, both of which have near omnipresent wind similar to that in the Samoas and traditionally have had open and airy domestic architecture made of bamboo, wood, and palm fronds, with walls often omitted to enable easy passage of the trade winds in an extremely humid environment). Although systematic assessments of the LTBI burden among the population in American Samoa or in the Independent State of Samoa have not been published, the incidence of active TB disease reported in both jurisdictions is consistently low by any standard, but especially for a Pacific Island population.

The combination of trade winds and relatively wall-less domestic architecture has been by no means the sole contributor to the lower rates of TB in the Samoas compared with Pacific Island groups elsewhere in Polynesia and in Micronesia and Melanesia, which have differing environmental conditions, demographics, and traditional architecture that more customarily had a greater presence of walls. However, in the Samoas, the relatively constant trade winds and the basic traditional architectural form of the fale might have significantly contributed to the low burden of TB observed in their populations today.
